# Site-Specific PEGylated Adeno-Associated Viruses with Increased Serum Stability and Reduced Immunogenicity

**DOI:** 10.3390/molecules22071155

**Published:** 2017-07-11

**Authors:** Tianzhuo Yao, Xueying Zhou, Chuanling Zhang, Xiaojuan Yu, Zhenyu Tian, Lihe Zhang, Demin Zhou

**Affiliations:** State Key Laboratory of Natural and Biomimetic Drugs, School of Pharmaceutical Sciences, Peking University, 100191 Beijing, China; bayerce@163.com (T.Y.); glacia418@163.com (X.Z.); htyzyxj2003@126.com (X.Y.); fashankc@163.com (Z.T.); zdszlh@bjmu.edu.cn (L.Z.)

**Keywords:** adeno-associated virus 2, PEGylation, genetic code expansion, selective conjugation

## Abstract

Adeno-associated virus (AAV) is one of the most extensively studied and utilized viral vectors in clinical gene transfer research. However, the serum instability and immunogenicity of AAV vectors significantly limit their application. Here, we endeavored to overcome these limitations by developing a straightforward approach for site-specific PEGylation of AAV via genetic code expansion. This technique includes incorporation of the azide moiety into the AAV capsid protein followed by orthogonal and stoichiometric conjugation of a variety of polyethylene glycols (PEGs) through click chemistry. Using this approach, only the chosen site(s) was consistently PEGylated under mild conditions, preventing nonselective conjugation. Upon a series of in vitro examinations, AAVs conjugated with 20-kD PEG at sites Q325+1, S452+1, and R585+1 showed a 1.7- to 2.4-fold stability improvement in pooled human serum and a nearly twofold reduction in antibody recognition. Subsequent animal research on Sprague Dawley rats displayed a promising 20% reduction in antibody inducement and a higher virus titer in the blood. Together, our data demonstrate successful protection of an AAV vector from antibody neutralization and blood clearance, thereby increasing the efficiency of therapeutic gene delivery.

## 1. Introduction

Adeno-associated virus (AAV) is a single-strand DNA parvovirus with a 4.7-kb genome composed of the rep and cap genes flanked by inverted terminal repeats (ITRs) and packaged within an icosahedral viral capsid protein made up of three structural proteins VP1–3 that are encoded by the cap gene [1]. As a significant component of viral vectors used in gene therapy, AAV has many advantages, including its nonpathogenic nature [2,3], long-term stable gene expression [4,5,6,7,8], and the ability to infect both dividing and non-dividing cells [9,10,11]. Among all identified serotypes, AAV-2 is currently the best characterized and most commonly used vector in clinical trials. Utilized as a tool for gene therapy, recombinant AAV (rAAV) vectors have been constructed by removing endogenous viral genes and inserting expression cassette(s) between the flanking ITRs, thus carrying exogenous genes into target cells [12].

Despite its high potential for gene therapy, pre-existing immunity towards AAV remains a major obstacle for its application. Indeed, approximately 80% of the population is seropositive for antibodies against AAV-2 [13,14,15], and neutralizing antibodies to AAV-2 were the most prevalent antibodies detected compared to other serotypes [16]. Moreover, even quite low levels of antibodies can prevent successful transduction [17]. Administration of large amounts of AAV vectors, which is required to overcome barriers such as preexisting neutralizing antibodies and proteolytic enzymes, frequently recalls pre-existing memory T cells (e.g., AAV-specific CD8+ T cells) that are also induced by natural infections [18]. All of these factors may significantly limit effective gene transfer and ultimately lead to the failure of gene therapy.

To alleviate the neutralization effect of antibodies against AAV vectors, especially those that target capsid proteins, several approaches have been used, including the use of alternative serotype vectors (AAV1–10) [16,19], different routes of administration [20], immune suppression [21], capsid engineering, encapsulation [22], and even use of an empty capsid mutant as an antibody decoy [23]. Recently, developing a structure-guided approach was also a good choice, which means to evolve AAV variants with altered antigenic footprints that cannot be recognized by preexisting antibodies [24]. Nowadays, one direct way to protect the viral vectors from neutralization is to shield the capsid surface, especially the sites targeted by antibodies, with polymers that have low immunogenicity, like polyethylene glycols (PEG). PEGylation is a well-established technique for improving the pharmacokinetics and pharmacodynamics of protein pharmaceuticals [25] and has been widely investigated in viral vector protection, including in adenovirus production [26,27]. Previous studies have used AAV PEGylation to improve gene transfer or to protect the virus from antibody neutralization by covalently conjugating PEG to specific amino acid residues distributed over the capsid protein. In one study, Lee et al. conjugated different sizes of PEG chains with rAAV-2 viral lysine residues at various ratios, finding that only when used within a narrow range, PEG moderately protects AAV from neutralizing serum and does not compromise viral infectivity; otherwise, viral infectivity was significantly reduced. More specifically, rAAV conjugated to PEG 2000 showed a 2.3-fold increase in transduction efficiency at a ratio of 1000:1 in the presence of neutralizing antibodies [28]. In another study, Le et al. modified rAAV-2 capsids with PEGs activated by cyanuric chloride (CCPEG), succinimidyl succinate (SSPEG), and tresyl chloride (TMPEG) and demonstrated that AAV conjugated with SSPEG was susceptible to neutralizing antibodies, whereas TMPEG afforded the best protection from neutralization both in vitro and in vivo without compromising transduction efficiency [29]. Nevertheless, to covalently conjugate activated PEG molecules to reactive amino acids may result in random modification at unexpected sites or regions because of the uncontrollable distribution of reactive amino acids, which not only means that optimization by subjective selection of modifying sites is limited but also may negatively affect infectivity or other biological functions of the virus. As a result, precise site-specific PEGylation is needed for further optimization based on the structural-functional relationships of AAV vectors.

**Figure 1:**
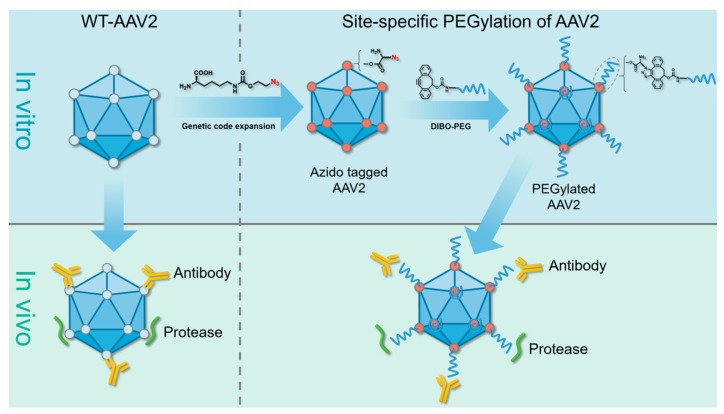
Diagram of precision PEGylation of adeno-associated virus 2 (AAV-2) to protect against recognition and degradation by neutralizing antibodies and proteases. The AAV-2 vector was site-specifically labeled with azide-bearing amino acids and then modified with polyethylene glycols (PEGs) via a bioorthogonal reaction to achieve preferable serum stability.

In this study, we used a straightforward and precise strategy of genetic code expansion [30,31] for PEGylation of recombinant AAV-2 vectors. Genetic code expansion means to genetically insert or change a natural codon to a stop codon that encodes an unnatural amino acid (UAA). In this study, we used a lysine mimic called Nε-2-azideoethyloxycarbonyl-l-lysine (NAEK), an azide moiety, at a chosen site of a protein [30,31,32]. Our approach resulted in the accurate modification of the capsid protein at a series of chosen sites, which protected the virus from pre-existing neutralizing antibodies and increased its serum stability (Figure 1).

## 2. Results 

### 2.1. Site-Specific PEGylation of VP1 Proteins and rAAV-2 Vectors via a Bioorthogonal Reaction

**Figure 2:**
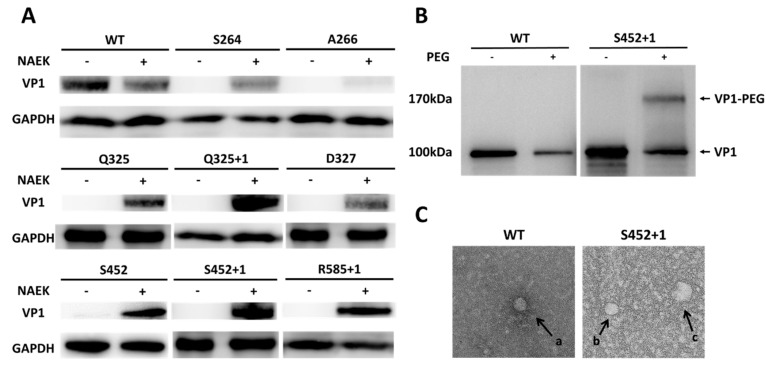
Feasibility of site-specific PEGylation of AAV capsid protein VP1 and AAV vectors by incorporation of NAEK (Nε-2-azideoethyloxycarbonyl-L-lysine, an azide moiety). (**A**) Analysis of NAEK-dependent expression of VP1 proteins by Western blotting. GAPDH was used as an internal control; (**B**) Western blotting analysis of site-specific PEGylation of VP1 proteins with the S452+1 mutant chosen as a representative; (**C**) Transmission electron microscope (TEM) images of naked or PEGylated rAAV2 vectors (a, b: unmodified particles; c: particle modified with 20-kD PEG).

We began by investigating if the amber suppressor with the orthogonal MbPylRS/MbtRNA_CUA_ pair was compatible for the expression of the AAV capsid protein VP1. Briefly, we chose the sites S264, A266, Q325, Q325+1, D327, R447, S452, S452+1, G453, G453+1, R459, E548, E548+1, S578, R585+1, N587, S662 for consideration (note: Q325 means the replacement of NAEK at this site, Q325+1 means the incorporation of NAEK between site 325 and 326). These sites were chosen after considering their tolerance, location, and potential functions, which were selected to avoid involvement in host cell recognition, receptor binding, or host–virus membrane fusion [33]. We specifically chose Q325+1, S452+1, G453+1, E548+1 and R585+1 as insertion sites because they are all distinct surface-exposed sites like N587+1, which site showed better performance than N587 in our previous work [34]. Additionally, the sites (R447, G453, G453+1, R459, E548, E548+1, S578, N587, S662) had already been proved successfully expression of NAEK-incorporated and NAEK-dependent VP1 proteins in our previous work [34]. Then, the VP1-FLAG-pcDNA 3.1(+) vector was directly site-mutated with the amber codon (TAG) inserted into the following sites: S264, A266, Q325, Q325+1, D327, S452, S452+1, R585+1. Next, the mutant plasmids were separately examined for their ability to express full-length VP1 with NAEK, an azide-containing unnatural amino acid that could be added to the peptide at the amber codon location with the help of an NAEK-specific orthogonal tRNA/aminoacyl-tRNA synthetase (tRNA/aaRS) pair. Western blot analysis showed that all mutant plasmids could generate full-length VP1 protein with a FLAG tag. When FreeStyle™ 293-F Cells were transfected and maintained in the presence of NAEK, the expression level of the different site-mutated VP1 proteins varied compared to that of wild-type VP1 (WT-VP1). In contrast, groups cultured without NAEK did not produce full-length VP1 because the amber codon would stop the translation process. As a control, WT-VP1 proteins were shown to be successfully expressed in either the presence or absence of NAEK owing to the absence of an amber codon in its sequence, as shown in Figure 2A. These results indicate that NAEK can be site-specifically incorporated into the AAV capsid protein VP1 via the strategy of genetic code expansion.

Additionally, to determine whether our synthetic DIBO-PEG (4-dibenzocyclooctynol-PEG) molecules could be successfully conjugated to NAEK-bearing VP1 protein, Western blot assays were performed. As shown in Figure 2B, a PEGylated protein band was detected after incubating the NAEK-bearing VP1 protein (S452+1 shown as a representative) with 10-kD DIBO-PEG. However, WT-VP1 incubated with 10-kD DIBO-PEG yielded no PEGylated band owing to the absence of NAEK. In addition, to directly view this modification on rAAV2 particles, WT rAAV2 and NAEK-containing rAAV2 were produced (S452+1 shown as a representative), purified, and incubated with 1 mM 20-kD DIBO-PEG at 4 °C for 2 h for observation under a transmission electron microscope (TEM) (Figure 2C). The images show that the unmodified particles have an average size of 20–30 nm, whereas the modified mutant particles have the larger size of approximately 40–60 nm, with an irregular edge. In the control, no modified particles could be detected in WT rAAV2 samples after incubation. These results indicate that AAV vectors labeled with NAEKs retained their structure after being modified with DIBO-PEG molecules.

### 2.2. Successful Production of Site-specific Mutant rAAV2 Particles and Their Structural-Functional Relationships

After determining the compatibility of NAEK insertion and site-specific PEGylation of VP1 proteins and rAAV2 vectors, we examined whether incorporation of NAEK at the chosen sites affected recombinant AAV packaging and infectivity by comparing the normalized virus genomic titers and the normalized ratios of functional titers (as measured by transgene expression) to genomic titers.

**Figure 3:**
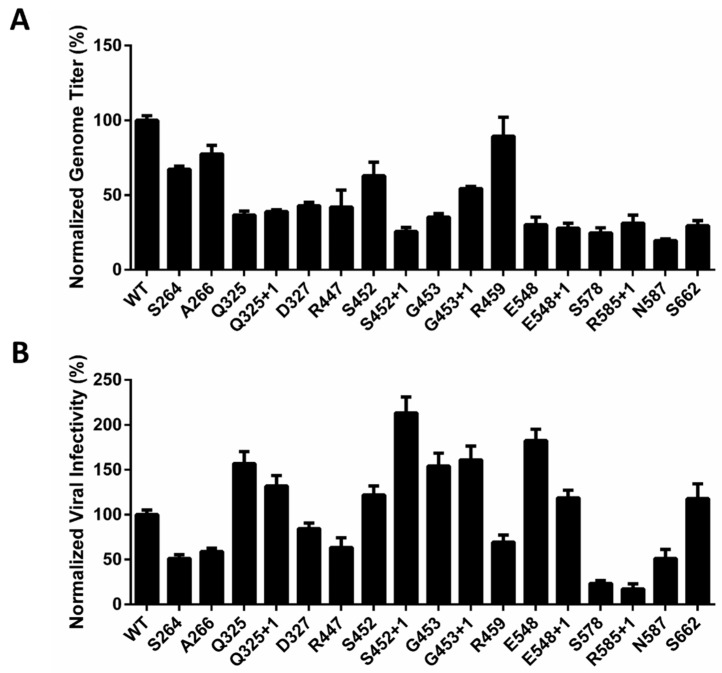
Comparing the effect of NAEK incorporation at different sites on the production and infectivity of rAAV2 vectors. (**A**) Viral packaging efficiency of site-specific NAEK rAAV2 mutants was measured by qPCR as the number of packaged genome copies/mL and normalized to that of the wild type; (**B**) The viral infectivity of site-specific NAEK mutants of rAAV2 vectors, which was calculated as the ratios of functional titers (transgene expression detected by luciferase assay on HT-1080 cells) to genomic titers and finally normalized to that of the wild-type group. Data represent the average values and standard deviations from triplicate experiments.

The production method for site-specific mutated rAAV-2 was based on a previous study from our lab [34]. As previously described, all AAV-293 cells were co-transfected with pAAV-RC-TAG, pHelper, pAAV-luc, and pPylRS/tRNACUA in the presence of 1 mM NAEK in the medium. The rAAV-2 particles were harvested after 72 h and isolated from cell debris and then directly quantified by real-time qPCR, as shown in Figure 3A. The relative genomic titer for S264, A266, S452, G453+1 and R459 were >50% of the WT-AAV genomic titer, suggesting a high tolerance for NAEK at these sites. In displaying NAEK at sites Q325, Q325+1, D327, R447, S452+1, G453, E548, E548+1, N587 and S662, approximately <50% of the genomic titer was maintained, indicating that NAEK insertion at these sites has some effect. These results reveal that some sites were comparatively tolerant to precise engineering by NAEK insertion.

Later, we quantified the functional titers of these mutant NAEK-carrying rAAV2 by luciferase analysis (Figure 3). The viral infectivity was measured by the ratios of functional titers to genomic titers, aiming to clarify whether the incorporations of NAEKs among these sites affected the virus infectivity. We found that mutants at sites Q325, Q325+1, S452, S452+1, G453, G453+1 E458, E548+1, and S662 showed a 1.0–2.0-fold increase in viral infectivity compared to that of wild-type AAV-2. This suggests that the NAEKs displaying on the viral surface can increase the viral infectivity of these mutants. The relative viral infectivity resulting from mutations at sites S264, A266, D327, R447, R459, S578, R585+1, and N587 showed a 10–80% reduction compared to that of wild-type AAV-2 indicating that emergence of NAEKs at these sites resulted in a greater loss in infectivity.

### 2.3. Optimized Sites for rAAV2 PEGylation

**Figure 4:**
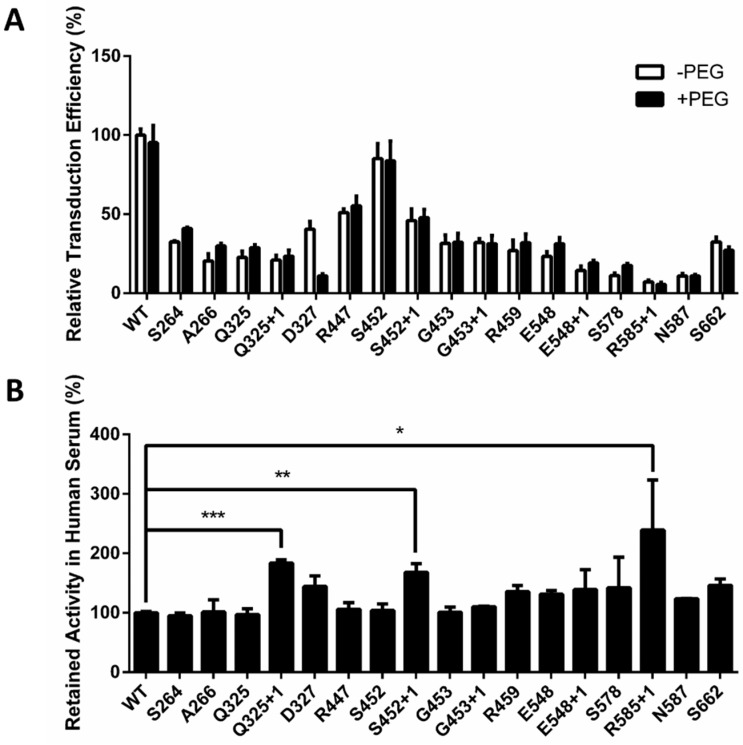
The effect of PEGylation on rAAV2 infectivity and stability in human serum. (**A**) Effect of PEGylation on in vitro transduction efficiency of rAAV2; (**B**) Effect of site-specific PEGylation on rAAV2 stability in human serum in vitro. PEGylated/naked rAAV2 vectors were incubated in pooled human serum/PBS and then used to infect HT1080 cells, as measured by an increase in fluorescence. The retained activity was calculated by an equation described in Section 4.8 and normalized to that of the wild-type control. All quantitative data shown are average values with standard deviations from triplicate experiments. A two-sample *t*-test was used to determine the statistical differences among the groups at the same time point. *** *p* < 0.001, ** *p* < 0.01, * *p* < 0.05.

Since it has been reported that PEG coverage may compromise the transduction efficiency of virus vectors in vivo [28], a pilot study was first initiated to evaluate the effect of PEGylation using our site-specific modification method. First, HT1080 cells were incubated with naked rAAV2/10k-PEGylated rAAV2 containing a firefly luciferase gene. Then, the fluorescence signal generated by successfully infected cells was examined. For most sites, PEGylation did not undermine the gene transduction capability of AAV, such as at Q325+1, S452+1, etc. (Figure 4A). In contrast, PEGylation of some sites caused a small reduction in transduction efficiency, such as at R585+1 and S662 (less than 20% effect). However, PEGylation at site D327 led to a nearly 70% loss of transduction efficiency. As a negative control, WT-rAAV2 was not PEGylated due to the absence of the unnatural amino acid and showed no difference whether co-incubated with PEG molecules or not. These results reveal that specific AAV PEGylation at most of these sites does not significantly diminish transduction efficiency.

To further select modification sites that provide optimal protection of the rAAV2 vectors, we examined the serum stabilities of the PEGylated AAV2 vectors. After co-incubating with healthy pooled human serum (diluted 1/500) at 37 °C for 30 min, the infectivity of the PEGylated vectors was measured. We found that a single site-specific PEG modification at most sites did not improve the stability of rAAV2 vectors as they rapidly lost their gene transduction efficiency, similar to that observed for naked rAAV2 vectors (Figure 4B). However, PEGylation at three sites, Q325+1, S452+1, R585+1, showed a promising improvement in gene transduction activity—a 2.4-fold increase was observed for R585+1 compared to the wild-type vector. These results demonstrate that optimized sites for AAV PEGylation can be produced by our site-specific method.

### 2.4. Optimal PEG Length for rAAV2 PEGylation

**Figure 5:**
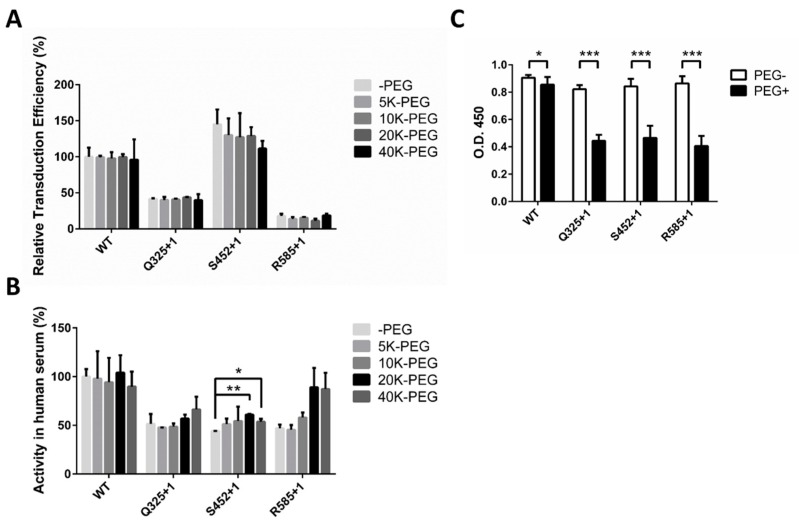
In vitro evaluation for PEGylated rAAV2 at sites Q325+1, S452+1, and R585+1 using 5-kD, 10-kD, 20-kD, and 40-kD PEG. (**A**) Transduction efficiency of rAAV2 modified with PEG of different molecular weights; (**B**) Effect of PEGylation size on the in vitro stability of rAAV2 in pooled human serum; (**C**) Site-specific PEGylation at Q325+1, S452+1, and R585+1 protects rAAV2 from recognition by antibodies in vitro. The affinity between anti-AAV polyclonal antibody and rAAV2 vectors modified/not modified with 20-kD PEG was determined by the enzyme-linked immunosorbent assay. Optical densities at 450 nm are shown. All quantitative data shown are average values with standard deviations from triplicate experiments. A two-sample *t*-test was used to determine statistical differences. *** *p* < 0.001, ** *p* < 0.01, * *p* < 0.05.

After identifying suitable sites for PEGylation using 10-kD PEG, we further examined whether 10-kD PEG was the optimal length for AAV PEGylation. Three mutant AAVs, Q325+1, S452+1, and R585+1, were chosen as representatives based on their performances in the human serum stability assay and were modified with PEG molecules of 5 kD, 10 kD, 20 kD, and 40 kD in size (Figure 5A). In examining the infectivity of these modified vectors, we found that modifying AAV with a larger molecular weight PEG resulted in a greater decrease in the transduction efficiency. When 20-kD PEG molecules were used, the decrease rate was 12% on S452+1, which increased to 23% when 40-kD PEG was used. This result reveals that larger PEG molecules could have a greater ability to cover recognition sites on the AAV capsid in host cells.

To further investigate the protective effect of different sizes of PEG molecules, the serum stabilities of rAAV2 vectors modified with 5 kD, 10 kD, 20 kD, and 40 kD PEG were tested. As shown in Figure 5B, the gene transduction retention rate increased in conjunction with the PEG molecular weight. For example, the 20-kD and 40-kD PEG-modified S452+1 mutant showed a statistically significant increase in stability compared to the unmodified one, and the R585+1 and Q325+1 mutants showed a similar, but not statistically significant, trend towards increased stability. However, modification with 20-kD and 40-kD PEG showed no significant difference among the three sites, indicating that there was a limit to the protective effect possible by simply increasing the PEG size. Based on the viral infectivity and serum stability results, 20-kD PEG was determined to be the most suitable length for AAV PEGylation.

### 2.5. Decreased Antibody Recognition of PEGylated rAAV2 In Vitro

Given that most people have already been exposed to AAV and consequently have antibodies against AAV [13,14,16], the clinical application of AAV vectors is greatly restricted. As a crucial index to evaluate protective efficacy, we investigate whether PEGylation of the AAV capsid could reduce antibody recognition. The enzyme-linked immunosorbent assay (ELISA) was conducted to evaluate the affinity between an anti-AAV polyclonal antibody purchased from Abcam (ab45482) and PEGylated rAAV2 vectors (20-K PEG) in vitro. We found that PEGylation at sites Q325+1, S452+1, and R585+1 resulted in an approximately 50% reduction in antibody recognition, as determined by a decrease in O.D. at 450 nm (Figure 5C). These results indicate that PEGylation on specific sites is able to protect rAAV2 vectors from recognition by antibodies in vitro.

### 2.6. In Vivo Verification of Improved Stability and Decreased Immunogenicity of PEGylated AAV2

**Figure 6:**
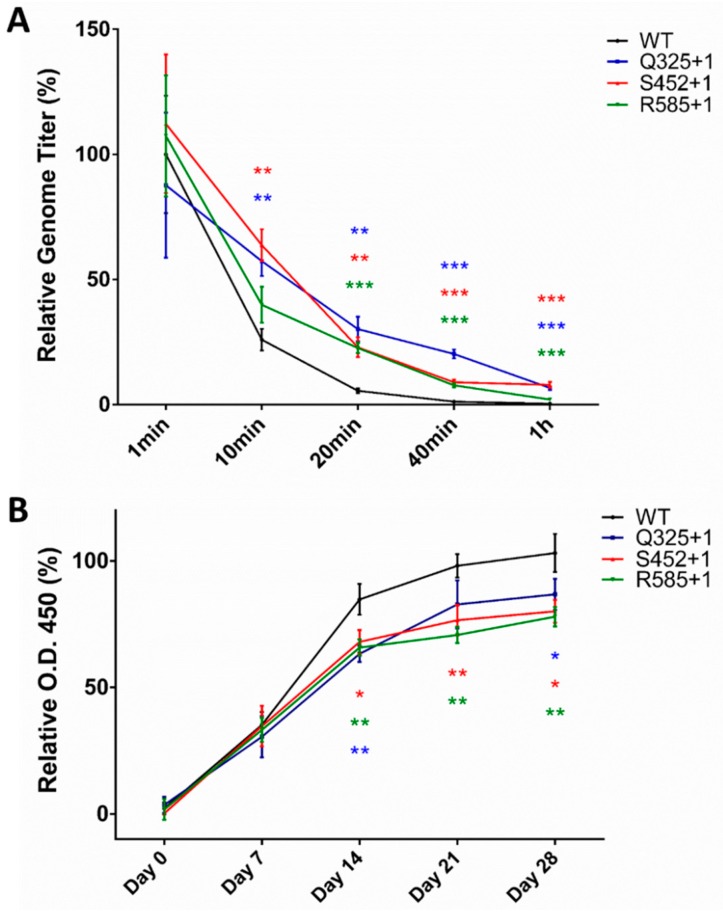
In vivo evaluation of PEGylated rAAV2 elimination in blood and antibody inducement using a Sprague Dawley rat model. (**A**) Blood clearance curve of PEGylated rAAV2. The genomic titer of retained rAAV2 in the blood at each time point was measured by quantitative PCR after a single dose injection of rAAV2, then normalized to that of wild type (WT) at 1 min; (**B**) Antibody induction experiment of PEGylated rAAV2 for a one-month period involving repeated stimulation. The level of VP1-specific antibodies in the blood at each time point was determined by an ELISA assay and presented in the form of normalized optical densities at 450 nm. All quantitative data shown are the average values with standard deviations from triplicate experiments. A two-sample *t*-test was used to determine the statistical difference among the groups at the same time point. *** *p* < 0.001, ** *p* < 0.01, * *p* < 0.05.

After in vitro evaluation of rAAV2 PEGylation, sites Q325+1, S452+1, and R585+1 were chosen as potential sites owing to their greater stability in human serum and nearly twofold reduction in antibody recognition. Moreover, 20 K was found to be the optimal size for PEGylation based on our results shown in Figure 4A,B. We then explored whether PEGylation could improve rAAV2 stability in the blood and lower the induction of antibodies in vivo. To determine this, a blood clearance curve for PEGylated rAAV2 was first determined. Sprague Dawley rats were injected with the same genome copy levels for WT rAAV2, three rAAV2 PEGylated mutants (Q325+1, S452+1, and R585+1 using 20-K PEG), or PBS. Blood samples were collected at a series of time points to determine the virus genomic titer level. As shown in Figure 6A, WT rAAV2 was quickly eliminated from the blood, retaining only 26% of the virus genomic titer after 10 min, 5% after 20 min, and the viral genome was nearly eliminated at 1 h. In contrast, S452+1 and Q325+1 kept 55–65% at 10 min, and the best performing mutant, Q325+1, retained 34% after 20 min and 23% after 40 min. These results indicate that PEGylation at specific sites can reduce the efficacy of rAAV2 elimination in the blood.

The following experiments examined whether rAAV2 PEGylation induces less antibodies in vivo. Sprague Dawley rats were injected every seven days for a total of four times with the same genome copy levels for WT rAAV2, three PEGylated rAAV2 mutants (Q325+1, S452+1, and R585+1), or PBS over the course of multiple immunizations. At the same time, blood samples were collected on the first day and every seven days to evaluate the level of antibodies by ELISA. In the WT group, antibodies targeting the VP1 protein were produced and increased rapidly in the blood after every stimulation resulting from rAAV2 injection (Figure 6B). Notably, in the PEGylated group, especially for R585+1, the ratio of the antibody production increase (presented as the slope of each curve) declined from day 14, and kept a lower level than that observed in the WT group until day 21. Additionally, the total content of antibodies induced by three PEGylated rAAV2 mutants all became less than wild-type from day 7, which remained to the final time point of the experiment, though only decreased to approximately 80% compared to that of the WT group on day 28, suggesting that PEGylation of capsid proteins can to some extent prevent rAAV2 from inducing as many antibodies as in the WT group.

## 3. Discussion

AAV vectors are a promising tool for gene therapy, whereas antecedent neutralizing antibodies pose a severe obstacle to their universal application. To overcome the challenge of pre-existing immunity, we developed a straightforward strategy for site-selective and site-specific PEGylation of AAV through genetic code expansion. Obviously, selection of the modification sites is quite significant in this site-specific strategy. To identify the appropriate site for PEGylation within the AAV capsid protein, we chose 17 candidate sites that were primarily located on the surface of the capsid protein. This determination was based on previous studies of AAV capsid protein modification and the crystal structure of AAV [35], taking the steric hindrance of NAEK insertion into consideration. The effect of PEGylation on each of the sites was then determined (Figure 4A) after confirming the successful production of NAEK-inserted AAV mutants. We found that the effect of PEGylation on virus infectivity depended on what site was modified. For sites D327, R585+1, and S662, PEGylation led to a reduction in transduction efficiency, suggesting that PEGylation at these sites may have protected part of the regions important for virus infectivity. Similarly, in the human serum stability assay, PEGylation at different sites displayed different levels of protection. On most sites, PEGylation led to a similar level of retained activity compared to that of wild type, suggesting nearly no protective effect. In contrast, sites Q325+1, S452+1, and R585+1 showed a promising improvement in stability, indicating that PEGylation at these sites may have protected part of the antibody binding region; thus, these sites were selected for subsequent in vivo research. Together, these results indicate that genetic code expansion is a potential approach to investigate the structural-functional relationships within the AAV2 vectors, which can also be regarded as a potential strategy to avoid detrimental effects on infectivity resulting from imprecise PEG conjugation.

Apart from the selection of modification sites, the molecular size of PEG also has an impact on virus infectivity and the level of protective efficacy. In this study of AAV site-specific PEGylation, we measured the effect of PEG size on AAV infectivity and stability in human serum, finding that larger PEG sizes can lead to better protection of AAV particles (Figure 5B, 20-K and 40-K PEG performed better than 5-K or 10-K PEG) but may also be accompanied by lower virus infectivity (Figure 5A, especially in S452+1). These results indicate that there may be a limitation in the protective effect offered by increasing the molecular size of PEG, probably because there is a balance between the sheltering of antibody-binding regions and the coverage of regions essential for virus infectivity, which depend on the location of each site. As a result, 20-K PEG was chosen for further study to determine its effect on antibody recognition (Figure 5C) and for use in animal research.

Though we optimized the selection of modification sites and the size of PEG in a series of in vitro investigations, the results of our in vivo tests were not consistent with our expectations. From the ELISA assay performed on Sprague Dawley rats (Figure 6B), we found that PEGylation at Q325+1, S452+1, and R585+1 can, to an extent, prevent rAAV2 from inducing as many antibodies as the wild-type group, with PEGylation at R585+1 showing the strongest protective effect. However, the reduction ratio observed was only up to 20%. On the one hand, modification at these sites may not be able to fully cover the antibody-binding region, indicating that additional thorough consideration of site choice is necessary; for instance, we could also screen sites near or within the antibody-binding region. On the other hand, a small percentage of unmodified particles within AAV samples may play a role in inducing more antibodies, which is consistent with TEM images showing that some naked particles can also be observed in the PEGylated rAAV2 samples. In addition, a Western blot analysis (Figure 2B) revealed the incomplete conjugation between DIBO-PEG and mutant VP1 at the protein level. Thus, the conditions used for PEG conjugation may need further optimization when transitioning from in vitro to in vivo experiments to reduce the immunogenicity of AAV vectors.

In conclusion, this research should lay a foundation for further studies and have clinical applications for protection of AAV vectors from antibody neutralization as well as in blood clearance, especially in patients with pre-existing AAV immunity. Our findings may also contribute to investigations of site-specific modifications and the structural-functional relationships of AAV. Given that our genetic code expansion approach is straightforward and offers high fidelity, this strategy could also be applied to other types of viruses. In addition, further studies should examine capsid-specific CD4+ and CD8+ T cell responses to PEGylated rAAV2, both in vitro and in vivo, to take into consideration the expanding research on AAV capsid-specific T-cell responses that participate in the loss of rAAV2 vector expression.

## 4. Materials and Methods 

### 4.1. Cell Lines, Antibodies and Reagents

AAV-293 and HT-1080 cells were purchased from the Type Culture Collection of the Chinese Academy of Sciences (Shanghai, China) and cultured in Dulbecco’s Modified Eagle’s Medium (Gibco, Carlsbad, CA, USA) supplemented with 10% fetal bovine serum (FBS; PAN-Biotech, Aidenbach, Germany) and 2 mM l-glutamine (Gibco), in an atmosphere of 37 °C containing 5% CO_2_. FreeStyle 293-F Cells (Gibco) were cultured in FreeStyle 293 Expression Medium (Gibco), in an atmosphere of 37 °C containing 8% CO_2_. Anti-flag mouse monoclonal Mab was obtained from TransGen Biotech (Beijing, China). The rabbit polyclonal antibody against AAV was purchase from Abcam Inc. (Cat no. ab45482, Cambridge, MA, USA). A 4-dibenzocyclooctynol-PEG (DIBO-PEG; 5 kD, 10 kD, 20 kD, and 40 kD) was obtained from Kaizheng Biotech (Beijing, China). Pooled human serum was purchased from Solarbio (Beijing, China). A human adeno-associated virus type 2 ELISA kit was obtained from Bluegene (Shanghai, China).

### 4.2. Plasmid Construction

The helper-free recombinant adeno-associated virus packaging system was purchased from Agilent Technologies (Santa Clara, CA, USA) and contained the three expression vectors pAAV-RC, pHelper, and pAAV-MCS. The pAAV-LUC vector had a firefly luciferase gene inserted into the multiple cloning site of pAAV-MCS to serve as a reporter and also contained a CMV promoter and ITR sequence. The pAAV-GFP vector, which contains the GFP gene, was generated in the same way. The pAAV-RC vector contains rep and cap genes encoding AAV replication and capsid proteins, respectively. The pAAV-RC-TAG vectors were produced using the QuickChange Lightning site-directed mutagenesis kit (Agilent Technologies) to introduce site-specific TAG mutations in the sequence of pAAV-RC. The pHelper vector provides adenovirus E2A, E4, and VA genes. The pPylRS/tRNACUA vector encoding orthogonal amber suppressor aminoacyl-tRNA synthetase/tRNACUA pairs was donated by Dr. Peng Chen (College of Chemistry, Peking University).

### 4.3. rAAV2 Production and Purification

AAV-293 cells were cultured at a density of 70% and transiently cotransfected with four plasmids: pAAV-LUC, pAAV-RC-TAG, pHelper and pPylRS/tRNACUA at a ratio of 1:1:1:2 using polyethyleneimine (PEI; Polyscience, Sigma-Aldrich, St. Louis., MO, USA). The medium was replaced with fresh medium containing 1 mM NAEK after a 6-h incubation. Next, the transfected cells were cultured for an additional 72 h prior to harvesting. To release rAAV2 particles, the transfected cells were lysed by four repetitions of thaw-and-freeze cycles. Finally, the cells fractions were centrifuged and discarded. The isolation and purification of rAAV2 from the cell lysis fraction by anion-exchange column chromatography was performed with AKTA pure as previously reported [36]. Further purification by ultracentrifugation at 43,000 rpm at 20 °C for 24 h using an Optima XPN-100 ultracentrifuge (Beckman Coulter, Inc., Brea, CA, USA) was performed in order to reduce the amount of non-viral proteins and improve the purity of the virus samples.

### 4.4. Quantification of Viral Genomic Titer by Real-time qPCR

The quantification of rAAV2 gene copy levels was performed using an Agilent Mx3000P real-time qPCR (Agilent Technologies) with the following primer pair designed to specifically amplify the firefly luciferase gene: 5′-GTGTTGTTCCATTCCATCACGG-3′ (forward) and 5′-CCAGCAGCGCACTTTGAATC-3′ (reverse). The preparation of samples, the construction of a standard curve, and the PCR process were performed under the guidance of a previous study [37].

### 4.5. Detection of Gene Transduction 

HT1080 cells were cultured for 24 h in a 96-well plate one day prior to beginning the assay. On the next day, the density of the HT1080 cells was around 50%. Then, AAV samples were added at a series of concentrations. After a 2-h incubation, the viruses were replaced with fresh culture medium, and the cells were cultured for an additional 48 h. Next, the medium was discarded and the fluorescence generated by firefly luciferase expressed from successfully transfected cells was detected using the Bright-Flo luciferase assay kit (Promega, Madison, WI, USA). 

### 4.6. PEG Conjugation

The DIBO-PEG molecules obtained were manufactured with an acetyl group at one terminus to provide a modifiable site. Then, DIBO was added onto the terminal end of PEG to provide an alkyne. Next, the purified azido-labeled rAAV2 was incubated with DIBO-PEG (1 mM) at 4 °C for 2 h. The excess unreacted DIBO-PEG was removed by ultrafiltration using 100-kD Millipore Amicon Ultra-100 filters (Merck Millipore, Tullagreen, Carrigtwohill, Ireland).

### 4.7. TEM of Negatively Stained rAAV2 Vectors

Transmission electron microscopy (TEM) is commonly used to directly visualize the intact viral particles. Purified and PEG-conjugated rAAV2 with a concentration of 0.6 mg/mL was loaded on a glow-discharged holey carbon grid (1.0-μm hole size, 400 mesh) by inversion of the grid on a 4-μL drop of viral suspension. The grid was then stained three times by inversion of the grid onto a 3-μL drop of 2% uranyl acetate for 10 s. At last, the edges of the grids were gently touched to Whatman paper for drying the grids. The viral particles were visualized using a FEI Tecnai G2 F20 electron microscope (FEI Company, Hillsboro, OR, USA).

### 4.8. Human Serum Stability Assay

rAAV2 vectors were first conjugated or not conjugated to 20-kD PEG molecules, then incubated with 1/500 diluted pooled human serum or PBS separately at 37 °C for 30 min. Next, HT1080 cells were infected by these PEGylated/naked and serum/PBS-incubated rAAV2 vectors separately as described in Section 4.5. Finally, chemical fluorescence was detected and the retention rate of gene transduction was calculated as below. 

Retained activity in human serum = (d/c)/(b/a)(1)

The letters (a,b,c,d) in the equation represent chemical fluorescence: a, unmodified rAAV2, PBS incubated; b, unmodified rAAV2, serum incubated; c, PEGylated rAAV2, PBS incubated; d, PEGylated rAAV2, serum incubated.

For this assay performed on the PEGylated selected sites Q325+1, S452+1, and R585+1, gene transduction was detected and calculated by the method below, which differed from Equation 1 to show a comparison between the PEGylated and unmodified groups at each site.

Activity in human serum = d/c(2)

The letters (c,d) in the equation have the same meaning as indicated in Equation 1. 

### 4.9. rAAV2 ELISA

First, 100 μL of balancing buffer (0.2% BSA, 1% polyvinylpyrrolidone and 3.1 mM sodium azide in PBST) was added to 10 μL of every rAAV2 samples. A series of standard solution samples as well as a blank control were respectively added (100 μL each) into a blank ELISA plate. Then, 50 μL of enzyme-labeling solution was added and incubated at 37 °C for 1 h. After 5 repetitions of washing with ELISA wash solution and drying, 50 μL of 3,3′,5,5′-tetramethylbenzidine (TMB) color developer solution A and 50 μL of solution B were added sequentially and incubated at 37 °C for 10–15 min in the dark. Finally, 50 μL of reaction stopping solution was added, and the optical absorbance was measured at 450 nm.

### 4.10. Animal Research

The animal experiments were performed according to the Guide for the Care and Use of Laboratory Animals approved by the Biomedical Ethics Committee of Peking University (approval no. LA2016064). To determine the half-life of PEGylated rAAV2 in vivo, Sprague Dawley rats (male, six weeks old, weighing around 300 g, with n = 5 mice per group) were randomly grouped and the caudal vein injected with WT-rAAV2, PEGylated rAAV2 mutants, or 100 μL of PBS (the genome copy number of AAV was 1 × 10^11^), respectively. Blood samples were then collected at time points of 1 min, 10 min, 20 min, 1 h, 2 h, 4 h, and 24 h. The samples were centrifuged at 2000× *g* for 30 min to collect the serum, with 1% heparin added at a ratio of 1:10. The AAV genomic titer of every sample was examined by real-time qPCR as described in 4.4.

To examine the induced-antibody concentration, Sprague Dawley rats (same characteristics as described above) were randomly grouped and were injected with WT-rAAV2, PEGylated rAAV2, or 100 μL of PBS (the genome copy number of AAV was 1 × 10^11^). The injection was repeated every 7 days for a total of 4 times. Blood samples were collected on the first day and every 7 days subsequently, for a total of 5 times. The antibodies in the blood samples were determined by ELISA as described below.

First, 100 μL of VP1 protein (1 ng/μL) was used to coat the ELISA plate. In addition, 2 μL of rat serum samples were diluted in ELISA wash solution to a volume of 100 μL and added to the plate (as well as standard solutions and controls) and incubated at 25 °C for 1 h. After three washes, goat anti-mouse IgG-HRP was added (1:5000 dilution) and incubated at R.T. for 1 h. After three repetitions of washing and drying, 50 μL of TMB color developer solution A and 50 μL of solution B were sequentially added and incubated at 37 °C for 10–15 min in the dark. Finally, 100 μL of the reaction stopping solution was added, and the optical absorbance was measured at 450 nm.

### 4.11. Statistical Analysis

All the experiments were repeated three times. Data are shown as the mean ± standard deviation. The results were analyzed by two-sample *t*-test with a significance level of 0.05.

## 5. Conclusions

In conclusion, we demonstrate a novel application of genetic code expansion for site-specific PEGylation of AAV particles, which provides a straightforward approach for systematic exploration of the structure-function relationship of PEGylated AAV vectors. Using this approach, nonselective conjugation was prevented and site-selective PEGylated AAV could be consistently prepared. Based on our in vitro findings of improved stability in human serum, Q325+1, S452+1, and R585+1 were chosen as potential sites. Upon examination of PEGylated AAV carrying different sizes of PEG at different sites, we found that larger sizes of PEG may result in better protection of AAV particles as well as decrease infectivity. We found that 20-K PEG was the optimal size for modification of those three sites. Subsequent in vitro research on resistance to antibody recognition showed a twofold reduction resulting from PEGylation of these sites. Unexpectedly, animal research resulted in a nearly 20% decrease in antibody induction, with blood clearance assays showing a modest trend towards half-life augmentation. Although additional optimization is necessary in site selection and experimental methods, our data suggest that this technology has potential for future research in protecting AAV vectors from antibody neutralization.
